# Elements of Electro-Metallurgy, or the Art of Working in Metals by the Galvanic Fluid, &c.

**Published:** 1841-04

**Authors:** 


ART. VI.—*Elements of Electro-Metallurgy, or the Art of working in Metals by the Galvanic Fluid, &c.* By ALFRED SMEE.—*London,* 1841. 8vo, pp. 163.

ALTHOUGH this work scarcely falls within the class of those to which the pages of our Review are properly dedicated, yet the author being a surgeon, and his volume being likely to be more read and better appreciated by the members of the medical profession, than by any other class of persons, we are induced to notice it. There is something highly creditable in the fact, that many of the most ingenious and interesting discoveries in the arts and sciences have emanated from men, either actually engaged in medical practice, or who have devoted themselves to the study of one or other of the special branches of medicine, more especially of chemistry. Whether this has been a profitable course to themselves or not is another qnestion; but at all events, society has derived considerable benefit from their labours.

Our readers, and more especially those who have made chemistry a study, must have heard of the Electrotype, or the art by which impressions in copper may be taken by galvanism from medallions, casts, plates, or models; and many must have had an opportunity of witnessing the admirable perfection with which the most exquisite works of art, may be copied by these silent operations of nature. It is not our purpose to settle who is the original discoverer of this singular process; it is sufficient for us to state that Mr. Smee has, in our opinion, carried it far beyond any other individual; and that he has made it applicable to cases, which judging from published documents, we should hardly imagine the original discoverer could have foreseen.

The treatise before us is short, but it is full of practical information. The author states his experiments clearly, so that they may be easily repeated by others. There is enough matter to have made a volume three times the size, but he has wisely contented himself with condensing his language within the limits necessary to render his explanations intelligible. The subject discloses an entirely new department in chemistry; and we can sincerely recommend the book to those of our readers who take an interest in that science. In the next edition we would recommend Mr. Smee to pay more care to the general qualities of his style than he seems to have done in the present.

